# The Iowa Gambling Task: Men and Women Perform Differently. A Meta-analysis

**DOI:** 10.1007/s11065-024-09637-3

**Published:** 2024-03-11

**Authors:** Ludovica Zanini, Chiara Picano, Grazia Fernanda Spitoni

**Affiliations:** 1 Department of Dynamic and Clinical Psychology, and Health Studies, Sapienza University of Rome, Via degli Apuli, 1, Rome, Italy; 2 Department of Psychology, Sapienza University of Rome, Rome, Italy; 3 Cognitive and Motor Rehabilitation and Neuroimaging Unit, IRCCS Fondazione Santa Lucia, Rome, Italy

**Keywords:** Iowa Gambling Task, Decision-making, Risk-taking, Gambling, Executive functions, Neuropsychological assessment

## Abstract

The Iowa Gambling Task (IGT) was designed to assess decision-making under conditions of complexity and uncertainty; it is currently one of the most widely used tests to assess decision-making in both experimental and clinical settings. In the original version of the task, participants are given a loan of play money and four decks of cards and are asked to maximize profits. Although any single card unpredictably yields wins/losses, variations in frequency and size of gains/losses ultimately make two decks more advantageous in the long term. Several studies have previously suggested that there may be a sex-related difference in IGT performance. Thus, the present study aimed to explore and quantify sex differences in IGT performance by pooling the results of 110 studies. The meta-analysis revealed that males tend to perform better than females on the classic 100-trial IGT (UMD = 3.381*; p* < 0.001). Furthermore, the significant heterogeneity observed suggests high variability in the results obtained by individual studies. Results were not affected by publication bias or other moderators. Factors that may contribute to differences in male and female performance are discussed, such as functional sex-related asymmetries in the ventromedial prefrontal cortex and amygdala, as well as differences in sensitivity to wins/losses.

**Supplementary Information:**

The online version contains supplementary material available at 10.1007/s11065-024-09637-3.

## Introduction

Sex differences in psychology and neuropsychology have been a complex and controversial topic of increasing interest in recent years (see Halpern, 2013). Within these fields, sex differences have been explored in numerous areas, such as visuospatial abilities (Voyer et al., 1995), verbal abilities (Hyde & Linn, 1988), working memory (Voyer et al., 2017, 2021), personality traits (Grijalva et al., 2015), temperament (Else-Quest et al., 2006), and self-esteem (Kling et al., 1999). One of the most critical and relevant constructs, due to its impact on day-to-day life, is decision-making.

Decision-making is defined as the process of selecting the most appropriate action from a range of possible actions (Darby & Dickerson, 2017). Although its definition may seem simple, decision-making is an incredibly complex ability that entails a vast number of cognitive processes. Determining the appropriate action to implement in a specific situation requires a series of cognitive operations, starting from the initial motivation required to achieve a given goal. It is then necessary to focus one’s attention toward the chosen option, while simultaneously inhibiting any stimuli that might serve as a distraction or diversion from that goal. The ability to choose the most appropriate action is further influenced by a continuous evaluation of all the likely consequences of the alternative options. Lastly, it is crucial to constantly monitor the conditions under which one is operating; circumstances, consequences, and the goals themselves may change over time, especially in real-life settings (Darby & Dickerson, 2017).

The processes described above fall under the term executive functions*.* Other than decision-making, they also include impulse control, attention, cognitive flexibility, and working memory (Ozga et al., 2018). Traditionally, executive functions have been categorized into “cold” and “hot” functions. Cold functions are logic-based and emotion-independent (e.g., working memory), whereas hot functions are driven by emotions, motivation, and rewards (Ozga et al., 2018). Decision-making is usually considered a hot cognitive function, given that the individual is asked to make decisions that have potentially rewarding or harmful consequences (Salehinejad et al., 2021).

One of the most widely used tasks to measure decision-making is the Iowa Gambling Task (IGT; Bechara et al., 1994). The task was initially created to evaluate patients with ventromedial prefrontal cortex (VMPFC) lesions who often experience difficulties in decision-making. Specifically, these patients tend to be unaware of future consequences of their actions and are guided by the most immediate outcomes (Bechara et al., 1994).

In the original version of the task, subjects are given a loan of play money (i.e., $2,000) and four decks of cards of equal size and appearance. The subject is asked to select one card at a time from any one of the four decks for a total of 100 trials. Subjects are told that their goal is to maximize profit and that they are free to choose as many cards from each deck as they wish. Although each single card unpredictably yields wins or losses, the decks are structured so that two decks are ultimately advantageous (i.e., decks C and D) and two are disadvantageous (i.e., decks A and B). The decks differ in terms of both frequency and size of gains/losses; for example, deck A is characterized by smaller but more frequent losses, whereas the losses in deck B are less frequent but of bigger size. In this last example, the deck may seem more advantageous in the short term by yielding higher gains on single cards (i.e., $100 vs $50), but may also include cards with greater losses. Decks C and D ultimately yield higher long-term returns and are therefore considered to be the two advantageous decks (Bechara et al., 2000). Participants who do not learn to prefer the advantageous decks over the disadvantageous ones are considered to exhibit a decision-making impairment. The most common way of quantifying a preference for advantageous/disadvantageous decks is the net IGT score, originally used by Bechara and colleagues (2000). The net score is the difference between the total number of advantageous choices and disadvantageous choices, and is calculated as $$\left(\left(C+D\right)-\left(A+B\right)\right)$$. This index allows researchers to obtain an overall measure of task performance regardless of the currency used within the individual studies. In the 100-trial version, the net score ranges from − 100 to 100; positive values are indicative of a higher proportion of advantageous choices, and therefore of a better performance.

Interestingly, several versions of the IGT have been created and used throughout the years. However, research suggests that variables such as type of monetary reward used (i.e., real or fake money), nature of the instructions given to participants, and type of task (i.e., computerized or manual) do not play a significant role in task performance (Bowman & Turnbull, 2003; Bowman et al., 2005; Fernie & Tunney, 2006).

Although the IGT was originally constructed to study patients with VMPFC lesions, over the years it has become the standard for assessing decision-making (Dunn et al., 2006). Contrary to lesioned patients, healthy subjects tend to gradually increase the choices made from the advantageous decks throughout the progression of the task. However, a persistence in choosing riskier decks has been observed not only in patients with brain injuries, but also in a variety of disorders arising from poor impulse control, such as substance abuse and pathological gambling (Brand et al., 2005; Takano et al., 2010).

The IGT’s psychometric properties seem to suggest that it is a multi-trait task measuring functions relating to both problem-solving and the attentional domain (Gansler et al., 2011). Its relationship with other tasks intended to measure executive functions is rather complex. Although several studies have found that it does not seem to correlate with performance on the Wisconsin Card Sorting Test (WCST) (for a review, see Buelow & Suhr, 2009), other have linked perseverative errors in the WCST in normal adults with the risky decision-making component of the IGT (Brand et al., 2007). Furthermore, a study by Pacheco-Colón and colleagues (2019) assessing the measurement invariance across decision-making tasks found that the IGT, the Cups Task, and the Game of Dice Task all seem to measure a single construct, which the authors suggest may represent the ability to make optimal choices that maximize rewards in the presence of risk (Pacheco-Colón et al., 2019). Despite the complex relationship between the IGT and other executive function tasks, taken together the literature seems to indicate that IGT performance is related to various executive function and working memory related tasks (Buelow & Suhr, 2009). Regarding its ecological validity, the previously discussed results obtained in clinical populations suggest a link between this task and real-world clinically relevant risky behavior, such as substance abuse disorders and pathological gambling (Brand et al., 2005; Takano et al., 2010).

Other than identifying well-established differences in decision-making between healthy and clinical populations, recent literature has begun to explore individual differences in IGT performance within the healthy population. Past research has suggested that a factor that may play a role in determining IGT performance is sex. A difference between neurotypical males and females has been reported in several individual studies, suggesting that men tend to choose more advantageous cards than women in the standard 100-trial version of the IGT (for a review, see van den Bos et al., 2013). Although seemingly well-established, only a limited number of individual studies have observed this result, and a systematic assessment of this sex-related difference is lacking.

To further add to this complexity, sex-related differences in decision-making have also been approached by exploring factors and conditions that seem to differentially impact men and women’s performances. For example, acute stress induced through the Trier Social Stress Task has been found to disproportionally alter the performance of men and women (Van den Bos et al., 2009); higher cortisol levels in men were associated with a poorer performance, whereas an inverse relationship was observed in women, suggesting that women may make better long-term decisions than men under acute stress. Moreover, men seem to exhibit greater sensitivity to achievement-related tasks (such as the IGT) in terms of higher cortisol levels, whereas the opposite seems to be true for social rejection (Stroud et al., 2002).

Another factor that has been found to play a role in differentiating male and female decision-making abilities is endogenous testosterone levels. A study by Stanton and colleagues (2011) observed that higher testosterone levels were linked to riskier choices in both men and women; however, this effect was found to be more pronounced in women (Stanton et al., 2011). The decline in performance could be attributed to the suppressive effect of testosterone on cortical regions associated with self-regulation and impulse control, such as the medial orbitofrontal cortex, resulting in behavior characterized by heightened pursuit of rewards and diminished sensitivity to potential consequences (Mehta & Beer, 2010).

Lastly, trait anxiety also seems to differentially impact men and women’s decision-making abilities (de Visser et al., 2010). In men, low and high levels of trait anxiety have both been associated with an impaired IGT performance compared to those with medium levels. On the contrary, IGT performance in females seems to only be hindered by high levels of trait anxiety.

Nonetheless, despite the complexity of the literature on the topic, thus far the exploration of sex-related differences in IGT overall performance has been limited to individual studies using varying experimental paradigms and conditions, but a systematic assessment of the difference between men and women’s IGT performance in the healthy population is still lacking. The present meta-analysis therefore aimed to assess and quantify sex differences in IGT performance, hypothesizing that men and women would obtain significantly different IGT net scores in the classic 100-trial task.

## Method

### Literature Search and Study Selection

The current meta-analysis was performed according to the PRISMA guidelines for systematic reviews and meta-analyses (see PRISMA checklist included in Online Resource 1), and the selection process for suitable publications was organized according the four steps included in the PRISMA flow diagram.

Firstly, potentially eligible articles were identified via a predefined algorithm in two electronic databases: pubMED (https://www.ncbi.nlm.nih.gov) and psychINFO (https://www.apa.org/pubs/databases/psychinfo/). The following search algorithm was used: “Iowa gambling task” OR “igt.” The search was conducted on 15 January 2023 and was limited to English-language publications published in the prior 20 years; this was done in order to systematize an ample time frame in which the use of the IGT version of interest in the current meta-analysis has been highly prevalent. The second step involved the exclusion of all duplicates and the screening of titles and abstracts in order to exclude irrelevant studies. Subsequently, the eligibility of the remaining articles was assessed by applying the following inclusion and exclusion criteria. Inclusion criteria were (a) use of the original 100-trial version of the IGT with two advantageous and two disadvantageous decks of cards, (b) the inclusion of both male and female participants, and (c) the inclusion of healthy adult participants (i.e., over 18 years old). Exclusion criteria were (a) use of an alternative version of the IGT (e.g., different number of trials or decks), (b) review articles and/or meta-analyses without any new data, (c) case reports, (d) studies conducted exclusively on clinical samples, and (e) studies conducted exclusively on participants below 18 years of age. All of the aforementioned steps were conducted by two independent reviewers; upon disagreement regarding a study’s eligibility, reviewers discussed their view until a consensus was reached. Eligible articles which provided all of the necessary data were included in the present meta-analysis; if any necessary information was missing, corresponding authors were contacted via e-mail. Furthermore, authors of publications including multiple studies with independent outcomes were also contacted in order to retrieve the data and characteristics pertaining to each independent sample.

### Data Extraction

The following data was extracted from every included study or for a subsample of a study (e.g., if only a portion of the participants took part in the IGT or if healthy controls were compared to a clinical population): (a) author and publication year, (b) number of males and females, (c) mean age, (d) task version (computerized/manual), (e) monetary reward (real/not real), (f) study quality (0–7), (g) region in which the study was conducted (North America/South America/Europe/Asia/Africa/Oceania), (g) mean and standard deviation of the IGT total net score for males, and (h) mean and standard deviation of the IGT total net score for females. The IGT total net score is the difference between the number of advantageous and disadvantageous choices made throughout the 100 trials (i.e., $\left(\left(C+D\right)-\left(A+B\right)\right)$); a higher net score reflects a higher proportion of advantageous choices made by the participant throughout the task, and therefore a better performance.

The quality of the studies was assessed through an index derived from the Newcastle–Ottawa Scale (NOS) (Peterson et al., 2011) often used in meta-analyses and systematic reviews (e.g., Cruciani et al., 2021). The quality index ranges from 0 to 7 points; the criteria used to assess the quality of each study are specified in Online Resource 2.

### Data Analysis

Data analysis was performed using the software Jasp 0.18.0.0. All of the analyses were performed using random-effect models to account for variance caused by differences among participants within and between studies. Effect sizes were computed for each study in terms of Unstandardized Mean Difference (UMD) in order to facilitate the interpretation of the results. The Restricted Maximum Likelihood estimator was used to estimate between-study variance (Veroniki et al., 2016). The 95% confidence intervals (CI) for between-study variance were created using the *Q*-profile method, and the Hartung-Knapp adjustment was applied (IntHout et al., 2014). Heterogeneity between studies was assessed using *Q* and *T*^2^ statistics.

Moderator analysis was performed for the following variables: mean age, monetary reward (real/not real), publication year, sample size, study quality, task version (computerized/manual), and region (North America/South America/Europe/Asia/Oceania). All moderators were included within the same model to allow for the interpretation of the effect of each moderator while controlling for the remaining variables. Continuous moderators were evaluated using meta-regressions, whereas categorical moderators were entered as grouping variables in the effect size calculations.

Regarding publication bias, a funnel plot was generated and visually assessed for signs of asymmetry. Begg and Mazumdar’s rank correlations and Egger’s regression intercept were calculated to test for small-study effects (i.e., when smaller studies obtain different effects than larger studies), for which publication bias may be one of the possible causes (Egger et al., 1997). Finally, a step-weight function selection model approach was computed to test and adjust the estimated effect for potential publication bias (Iyengar & Greenhouse, 1988; Vevea & Hedges, 1995).

## Results

**Fig. 1:**
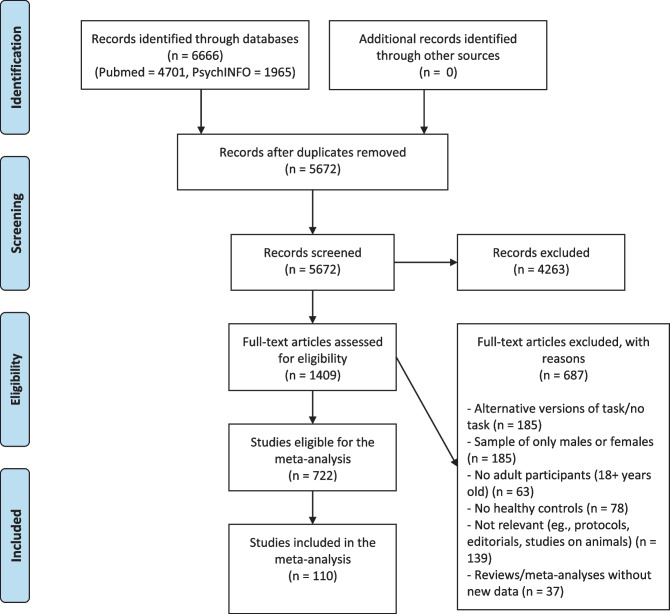
The diagram summarizes the study selection process in compliance with the PRISMA guidelines for systematic reviews and meta-analyses

The various steps of the selection process are summarized in Fig. 1. A total of 6666 articles were initially identified through the database search. After removing duplicates, the titles and abstracts of the remaining 5672 records were screened. A full-text assessment was then performed on 1409 articles by applying the aforementioned inclusion and exclusion criteria. Of these, 722 records were deemed eligible for inclusion. However, in order to perform the present meta-analysis, additional data had to be extracted (e.g., mean and standard deviations of the IGT net score for males and females); this information was often not reported within the publications. Thus, the necessary data was requested by contacting the corresponding author of each study. Among the 722 eligible studies that met all inclusion and exclusion criteria, 110 were ultimately included in the present meta-analysis.

Table 1 summarizes the characteristics and the data extracted from the 110 studies. Furthermore, details on the quality assessment indices obtained through the Newcastle Ottawa Scale are reported in Online Resource 3.

**Table 1:** Characteristics of the studies included in the meta-analysis, including extracted means and standard deviations used to compute effect sizes

**Studies (*n* = 110)**	**Sample size (females)**	**Mean age**	**Task version**	**Monetary reward**	**Study quality (0–7)**	**Mean net score (SD)**	
						**Males**	**Females**
Alameda-Bailén et al. (2018)	72 (19)	24.47	Computerized	Not real	6	−0.79 (32.80)	6.69 (24.47)
Aloi et al. (2020)	26 (18)	46.70	Computerized	Not real	5	3.80 (23.90)	17.10 (30.20)
Bangma et al. (2019)	50 (25)	39.14	Computerized	Not real	7	13.68 (36.92)	7.36 (37.20)
Barnhart et al. (2019)	140 (78)	19.28	Computerized	Not real	6	1.77 (28.36)	1.59 (25.14)
Birkás et al. (2015)	60 (28)	22.35	Computerized	Not real	6	−5.58 (24.99)	−7.36 (25.47)
Bolla et al. (2004)	20 (10)	30.05	Computerized	Real	5	25.20 (14.80)	–12.20 (25.30)
Bonnaire et al. (2022)	99 (38)	38.74	Computerized	Not real	7	16.98 (31.98)	11.53 (28.09)
Bouchard et al. (2012)	24 (2)	37.29	Computerized	Real	5	8.82 (22.24)	−7.00 (1.41)
Brunell and Buelow (2017) (study 1)	375 (231)	19.28	Computerized	Not real	6	10.72 (31.42)	−1.88 (23.29)
Brunell and Buelow (2017) (study 2)	231 (114)	19.29	Computerized	Not real	6	2.79 (27.45)	−5.51 (23.16)
Brunell and Buelow (2017) (study 3)	293 (165)	18.97	Computerized	Not real	6	4.71 (27.17)	2.43 (22.65)
Buelow and Barnhart (2017)	137 (82)	19.07	Computerized	Not real	6	8.19 (30.47)	−5.18 (24.90)
Buelow and Barnhart (2018)	93 (52)	19.26	Computerized	Not real	6	−0.07 (29.47)	2.62 (27.40)
Buelow and Blaine (2015)	390 (235)	18.86	Computerized	Not real	6	3.01 (28.70)	−0.77 (23.31)
Buelow and Brunell (2020)	244 (165)	18.63	Computerized	Not real	6	0.56 (32.93)	−8.50 (24.15)
Buelow and Suhr (2013)	91 (53)	19.04	Computerized	Not real	6	7.89 (28.17)	3.18 (26.01)
Buelow and Suhr (2014)	136 (88)	19.24	Computerized	Not real	6	5.92 (28.03)	1.95 (23.38)
Buelow and Wirth (2017) (study 1)	83 (43)	18.56	Computerized	Not real	6	2.60 (25.83)	2.19 (24.35)
Buelow and Wirth (2017) (study 2)	120 (60)	18.79	Computerized	Not real	6	−5.31 (23.82)	−4.63 (24.05)
Buelow et al. (2013) (study 1)	192 (118)	19.44	Computerized	Not real	6	4.63 (29.89)	0.09 (24.31)
Buelow et al. (2013) (study 2)	260 (149)	19.18	Computerized	Not real	6	2.14 (29.44)	−4.57 (23.68)
Buelow et al. (2015a)	216 (114)	19.24	Computerized	Not real	6	5.32 (27.55)	−1.27 (23.24)
Buelow et al. (2015b)	65 (35)	19.47	n.s	n.s	4	−0.32 (22.48)	4.26 (18.22)
Burke et al. (2011)	142 (81)	21.04	Computerized	Not real	4	9.70 (23.51)	8.32 (21.18)
Bø et al. (2016)	121 (62)	21.70	Computerized	Not real	7	27.88 (20.83)	29.82 (18.73)
Casey and Cservenka (2020)	32 (12)	19.25	Computerized	Not real	6	42.50 (15.99)	34.83 (30.98)
Clay and Parker (2018)	16 (7)	23.38	Computerized	Not real	5	13.33 (42.93)	5.29 (22.92)
Crane et al. (2013)	69 (25)	20.74	Computerized	Not real	7	45.59 (9.50)	45.60 (10.26)
Daurat et al. (2013)	20 (5)	50.25	Computerized	Not real	5	2.80 (25.30)	12.80 (56.30)
Delazer et al. (2016)	20 (11)	45.40	n.s	n.s	4	16.89 (24.23)	22.27 (24.46)
Demaree et al. (2010)	68 (35)	19.47	Computerized	Not real	6	13.49 (31.81)	9.82 (22.42)
Dingemans et al. (2019)	60 (52)	37.15	Computerized	Not real	7	2.96 (29.97)	16.25 (18.56)
Dreves et al. (2020)	182 (117)	19.64	n.s	n.s	6	49.11 (30.12)	31.59 (35.99)
Dreyer et al. (2022)	40 (19)	20.21	Computerized	Not real	6	2.38 (7.66)	1.92 (5.67)
Emery et al. (2020)	1295 (683)	44.63	Computerized	Not real	7	21.62 (29.81)	17.58 (28.89)
Farrell and Walker (2019)	112 (78)	42.20	Computerized	Not real	7	−4.73 (38.99)	2.91 (38.27)
Favieri et al. (2022)	29 (15)	24 (3)	Computerized	Not real	6	21.43 (28.38)	−6.11 (17.40)
Fernández et al. (2022)	26 (16)	33.81	Computerized	Not real	5	35.60 (17.80)	34.80 (19.80)
Gescheidt et al. (2013)	18 (7)	50.61	Computerized	Real	5	16.00 (25.98)	−2.86 (23.91)
Ghosh et al. (2021)	10 (8)	26.50	Computerized	Not real	5	31.00 (21.21)	16.79 (24.40)
Giustiniani et al. (2019)	20 (10)	38.70	Computerized	Real	5	4.24 (6.52)	3.60 (4.92)
Gkintoni et al. (2017)	102 (55)	36.63	Computerized	Not real	6	1.98 (54.01)	−3.69 (45.08)
Gullo and Stieger (2011)	44 (34)	22.52	Computerized	Not real	6	13.60 (38.42)	3.59 (30.92)
Hart et al. (2010)	213 (127)	19.42	Computerized	Real	6	21.33 (31.33)	8.76 (28.78)
Hayes and Wedell (2020a)	73 (54)	21.88	Computerized	Not real	6	16.63 (29.27)	−3.41 (30.31)
Hayes and Wedell (2020b)	64 (48)	20.05	Computerized	Not real	6	2.50 (22.02)	−3.92 (21.28)
Heilman and Miclea (2015)	48 (42)	21.39	Computerized	Not real	6	0.00 (16.54)	4.38 (20.3)
Hulka et al. (2014)	68 (22)	30.63	Computerized	Real	7	13.70 (24.1)	20.10 (29.10)
Icellioglu (2015)	90 (45)	47.90	Computerized	Not real	6	4.00 (13.26)	0.13 (17.53)
Kashyap et al. (2013)	75 (19)	26.60	n.s	n.s	4	5.25 (18.59)	4.84 (24.61)
Kim et al. (2009)	55 (26)	28.80	Computerized	Not real	6	15.24 (28.31)	12.77 (27.88)
Kobayakawa et al. (2008)	22 (9)	67.60	Computerized	Not real	5	5.54 (10.20)	4.00 (14.90)
Kräplin et al. (2014)	53 (21)	36.74	Computerized	Not real	6	23.91 (24.67)	13.69 (28.30)
Lage et al. (2013)	125 (75)	24.28	Computerized	Not real	7	7.36 (20.01)	9.18 (20.89)
Lai et al. (2023)	30 (13)	54.53	Computerized	Not real	7	30.06 (38.50)	9.85 (33.87)
Lake et al. (2020)	20 (8)	35.55	Computerized	Real	6	−10.83 (27.68)	−14.25 (13.96)
Lee et al. (2009)	33 (19)	29.00	Computerized	Not real	6	12.86 (31.90)	10.11 (30.87)
Leonello and Jones (2016)	52 (29)	19.60	Computerized	Not real	6	3.57 (44.31)	−1.38 (32.06)
León et al. (2020)	91 (50)	20.76	Computerized	Not real	6	4.39 (27.87)	0.08 (35.34)
Linhartová et al. (2020)	55 (35)	23.42	Computerized	Not real	7	23.70 (45.45)	20.41 (32.43)
Lovallo et al. (2014)	705 (396)	23.72	Computerized	Not Real	7	14.32 (26.72)	11.73 (23.12)
Lucas et al. (2021)	191 (151)	25.65	Computerized	Not real	6	11.82 (16.76)	9.31 (13.74)
MacLaren et al. (2022)	100 (65)	21.31	Computerized	Not real	6	24.40 (27.92)	24.25 (25.83)
Maddaluno et al. (2022)	434 (257)	44.57	Computerized	Not real	7	17.36 (31.04)	16.52 (30.60)
Martín-Ríos et al. (2022)	171 (97)	47.44	Computerized	Not real	6	1.81 (25.6)	−2.74 (27.5)
Massar et al. (2014)	31 (23)	23.20	Computerized	Not real	6	−1.75 (38.13)	−4.55 (22.50)
Maurage et al. (2018)	38 (9)	46.66	Computerized	Not real	6	30.28 (30.02)	6.89 (14.15)
Merchán-Clavellino et al. (2019)	29 (22)	22.31	Computerized	Not real	5	4.29 (17.37)	5.36 (18.30)
Meshi et al. (2019)	71 (44)	23.70	Computerized	Not real	7	14.00 (19.86)	15.82 (18.61)
Miu et al. (2012)	135 (118)	21.60	Computerized	Not real	6	10.23 (35.79)	8.34 (22.53)
Molins et al. (2021)	43 (32)	22.43	Computerized	Not real	6	7.45 (15.83)	6.63 (16.27)
Moniz et al. (2016)	30 (20)	42.43	Computerized	Not real	6	25.80 (7.75)	34.80 (4.26)
Müller et al. (2021)	44 (36)	22.44	Computerized	Not real	6	19.75 (24.92)	6.56 (22.40)
Namba (2021)	57 (33)	19.60	Computerized	Not real	6	4.91 (30.88)	−10.00 (23.00)
Nicholson et al. (2021) (study 1)	87 (48)	28.60	Computerized	Not real	6	−10.46 (26.48)	−6.00 (30.93)
Nicholson et al. (2021) (study 2)	89 (55)	27.80	Computerized	Not real	6	−1.27 (6.04)	−1.39 (6.92)
Obeso et al. (2021)	11 (8)	25.00	Computerized	Real	6	6.24 (23.95)	2.69 (22.54)
Olkoniemi et al. (2016)	60 (49)	23.60	Computerized	Not real	6	25.45 (20.36)	10.20 (25.59)
Olson et al. (2016)	55 (29)	30.65	Computerized	Not real	7	9.31 (35.37)	20.00 (34.42)
Oswald et al. (2015)	45 (18)	22.70	Computerized	Real	7	21.30 (26.80)	10.20 (27.30)
Ouerchefani et al. (2017)	34 (4)	39.03	Manual	Not real	6	20.60 (14.34)	16.50 (10.25)
Paz-Alonso et al. (2020)	18 (3)	63.00	Computerized	Not real	5	46.73 (13.28)	37.67 (17.79)
Penolazzi et al. (2013)	165 (85)	26.47	Computerized	Not real	6	14.35 (27.40)	6.27 (21.08)
Premkumar et al. (2010)	15 (2)	35.40	n.s	n.s	4	27.27 (44.24)	32.00 (16.97)
Psederska et al. (2021)	311 (143)	28.42	Computerized	Not real	7	4.22 (28.07)	4.11 (26.96)
Runyon and Buelow (2019)	97 (63)	18.56	Computerized	Not real	6	−0.32 (25.61)	5.14 (27.29)
Sánchez-Torres et al. (2013)	42 (25)	32.10	n.s	Not real	5	5.94 (24.90)	−5.12 (21.18)
Sebri et al. (2021)	30 (16)	28.83	Computerized	Not real	7	12.42 (15.71)	15.62 (26.08)
Seubert-Ravelo et al. (2021)	25 (8)	55.30	Manual	Not real	5	14.65 (20.87)	−5.38 (16.22)
Shukla et al. (2019)	14 (9)	22.50	Computerized	Not real	5	−9.20 (16.71)	−1.11 (29.11)
Simonovic et al. (2017)	29 (15)	22.24	Manual	Real	5	33.78 (15.84)	44.80 (17.35)
Singh (2016)	320 (160)	23.81	Computerized	Not real	6	3.06 (27.13)	0.89 (25.84)
Siqueira et al. (2022)	55 (36)	69.95	Computerized	Not real	6	4.74 (15.88)	−4.78 (17.21)
Stinson et al. (2018)	46 (11)	37.20	Manual	n.s	7	7.37 (25.34)	0.91 (23.94)
Stoltenberg and Vandever (2010)	188 (117)	22.55	Computerized	Not real	6	19.77 (28.59)	17.61 (28.00)
Tarantino et al. (2021)	260 (174)	34.10	Computerized	Not real	6	12.50 (43.00)	3.92 (32.20)
Tchanturia et al. (2012)	61 (41)	23.26	Computerized	Not real	6	20.80 (36.00)	14.68 (26.80)
Valentini et al. (2017)	26 (12)	26.15	n.s	n.s	3	43.29 (35.28)	36.33 (29.33)
Vila-Rodriguez et al. (2013)	138 (95)	21.80	Computerized	Not real	6	19.95 (25.97)	12.72 (30.31)
Villanueva-Moya and Expósitoo (2021)	53 (27)	21.77	Computerized	Not real	6	11.00 (27.96)	37.85 (20.46)
Vrshek-Schallhorn et al. (2013)	21 (7)	18.91	Computerized	Real	5	17.29 (26.06)	4.57 (22.71)
Webb et al. (2014)	65 (32)	30.15	Computerized	Not real	6	9.31 (35.36)	19.79 (34.32)
Werner et al. (2013)	29 (17)	23.83	Computerized	Not real	5	27.58 (28.00)	7.59 (33.60)
Yechiam and Telpaz (2013) (study 2)	130 (65)	23.50	Computerized	Real	4	28.77 (35.04)	14.58 (36.41)
Yechiam et al. (2008)	25 (16)	39.00	Computerized	Real	6	17.56 (27.27)	−0.13 (34.52)
Yechiam et al. (2016) (study 1)	58 (29)	24.35	Computerized	Real	6	5.17 (36.02)	5.07 (31.79)
Zhang et al. (2017)	133 (72)	20.49	Computerized	Not real	6	5.02 (12.97)	6.78 (9.78)
Zhang et al. (2022)	25 (12)	38.00	Computerized	Not real	6	6.77 (49.50)	3.50 (34.89)
Zouraraki et al. (2019)	114 (58)	31.83	Computerized	Not real	6	7.39 (25.75)	9.82 (20.59)
Zourakaki et al. (2020)	236 (113)	34.28	Computerized	Not real	6	6.26 (25.61)	7.12 (21.48)

**Fig. 2:**
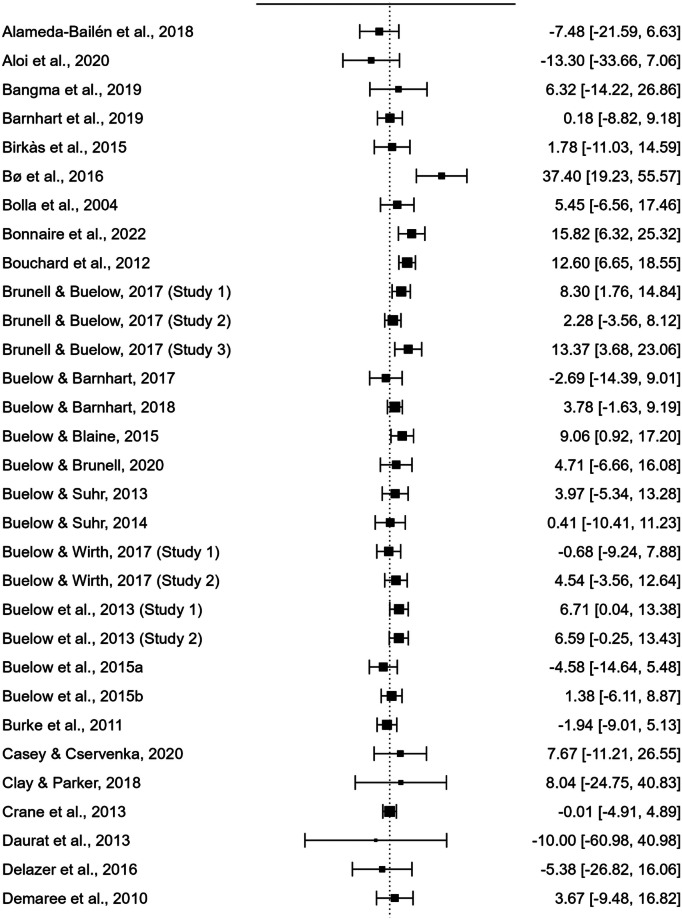

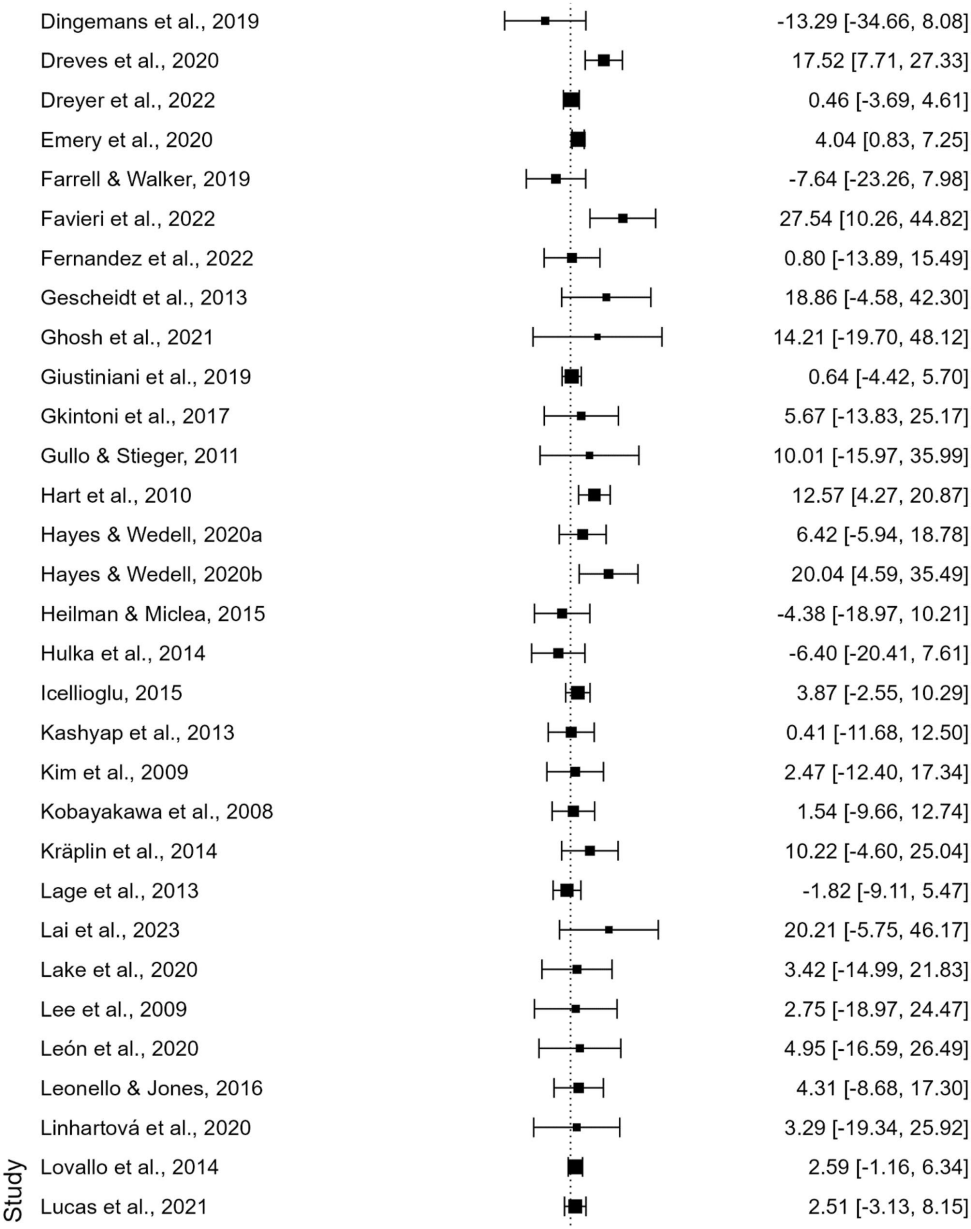

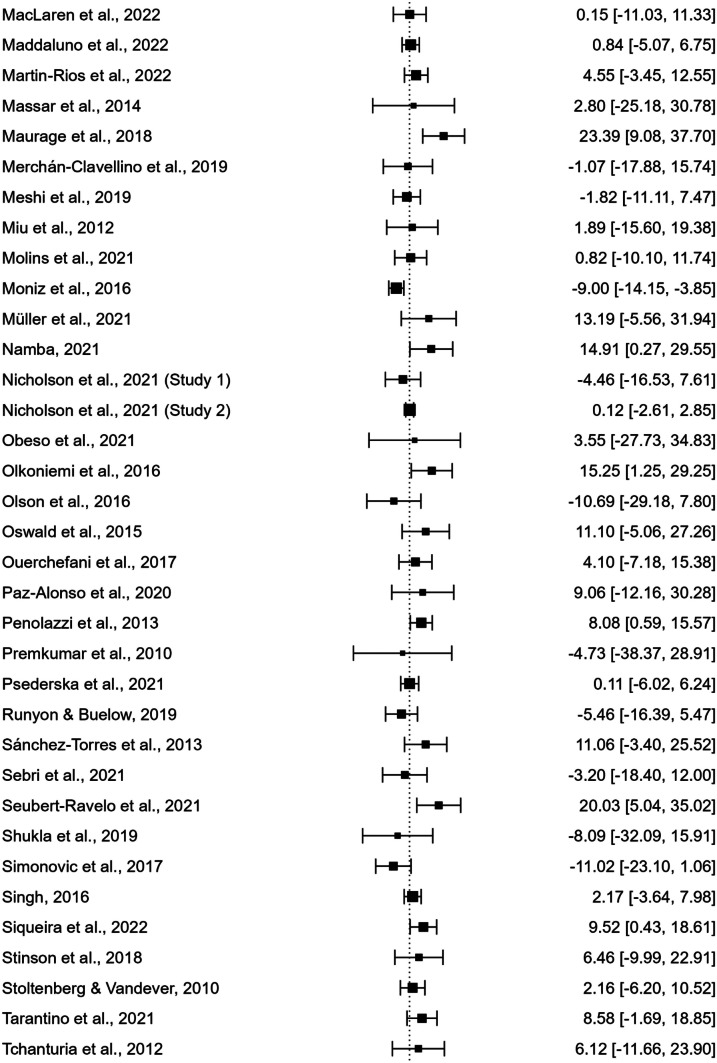

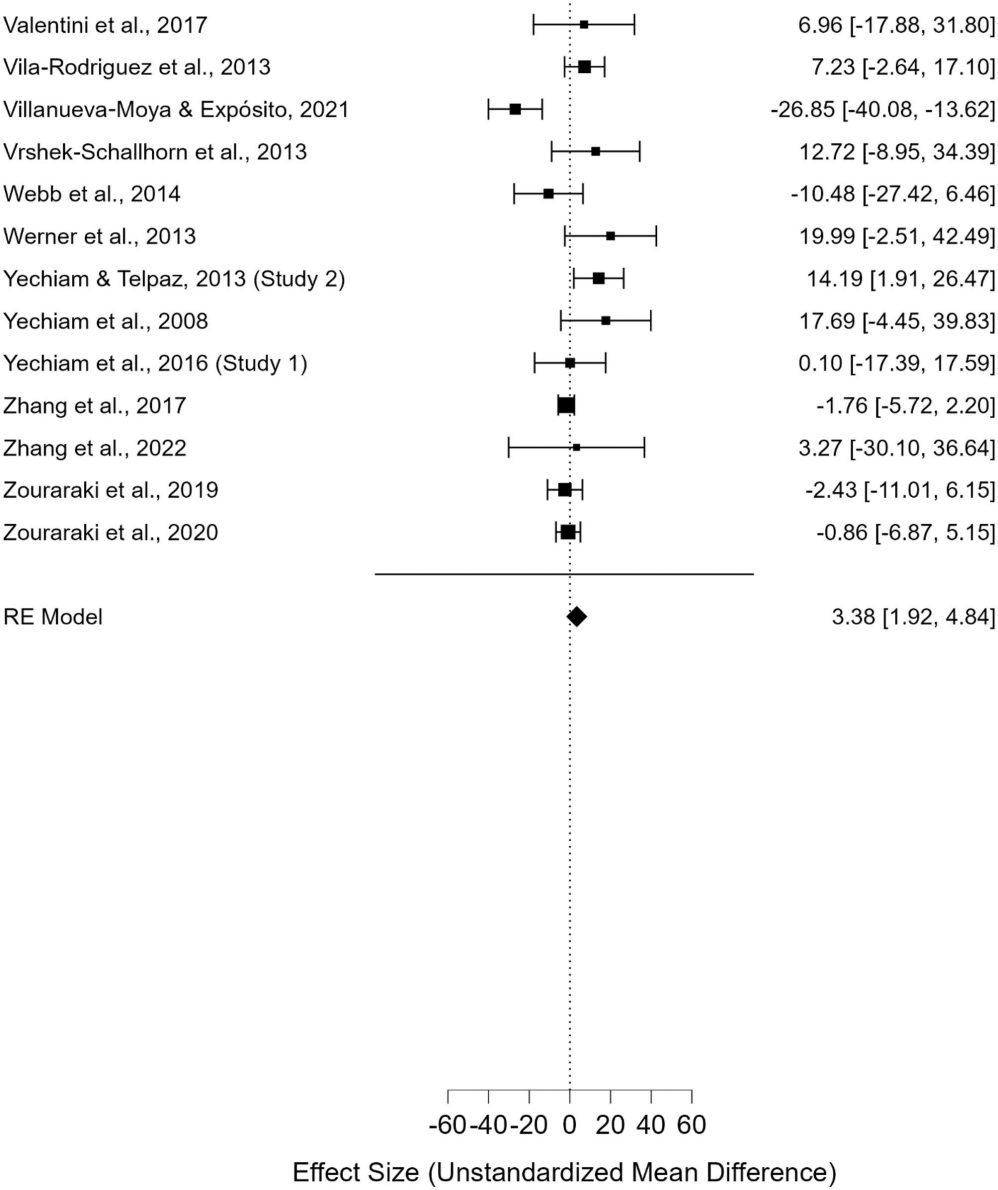
Forest Plot generated by Jasp 0.18.0.0. Black squares represent the effect size (ES) of each study included in the meta-analysis (derived from the comparison of male and female net IGT scores). The size of each square reflects the weighting of the single study within the pooled estimate. The horizontal lines (whiskers) represent the 95% confidence interval (CI) of each study result. The vertical line (*y*-axis), defined as the “line of null effect,” indicates the absence of differences between the two groups (males and females) in IGT performance. Studies depicted on the left and right of the line of null effect reported higher net IGT scores for females or males, respectively. The diamond at the bottom of the forest plot represents the overall effect size and confidence interval resulting from the combination of all individual studies

As displayed in the forest plot in Fig. 2, the meta-analysis revealed a significant difference between the IGT total net scores of males and females, with males performing better than females (110 studies; UMD = 3.381; *t*(109) = 4.592; standard error = 0.736; 95% CI [1.922, 4.841]; *p* < 0.001).

The heterogeneity across studies was also significant, as shown by the *Q* (*Q*(109) = 206.001; *p* < 0.001) and the *T*^2^ (*T*^2^ = 20.782; 95% CI [11.782, 48.921]) statistics.

**Fig. 3:**
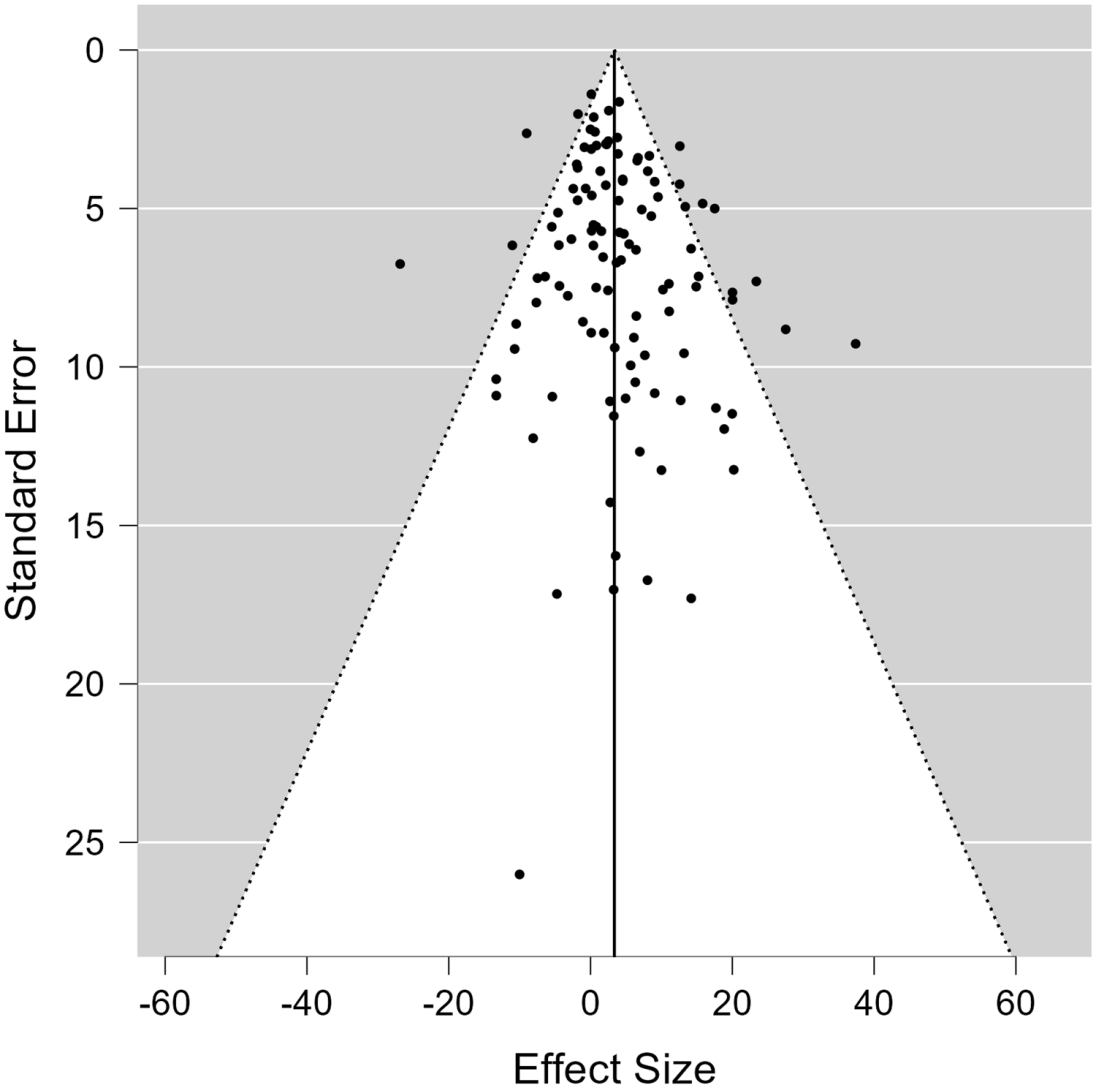
Funnel plot generated by Jasp 0.18.0.0. Each circle represents a single study. The *y*-axis represents the standard error of the estimated effect. The *x*-axis displays the result for each study (in terms of effect size). In the absence of publication bias, the distribution of the studies within the funnel plot is due to sampling variation alone and the plot resembles a symmetrical inverted funnel

Regarding publication bias, the funnel plot in Fig. 3 revealed no evidence of asymmetry; Kendall’s tau (*Z* = 0.081; *p* = 0.210) and Egger’s regression test (*t* = 1.570; *p* = 0.119) did not reveal any small-study effects, for which publication bias would have been considered one of the possible causes. Using a selection-model approach, assuming heterogeneity (*Q*(109) = 206.001; *p* =  < 0.001), no evidence of publication bias was observed (*X*^2^ = 0.491; *p* = 0.483). Furthermore, the publication bias-adjusted effect size remained significant (UMD = 2.747; standard error = 1.064; 95% CI [0.662, 4.832]; *p* = 0.010).

The results regarding the moderation analyses are summarized in Table 2; neither publication year, sample size, mean age, study quality, task version, monetary reward, nor region moderated the obtained results.

**Table 2:** Results of the moderation analyses

Moderator		Coefficient	*t*	*p*
Publication year		−0.070	−0.293	0.770
Sample size		0.001	0.313	0.755
Mean age		0.078	0.926	0.357
Study quality		−0.600	−0.399	0.691
Task version		−1.686	−0.306	0.761
Monetary reward		2.678	0.956	0.342
Region	Asia	0.402	0.072	0.942
	Europe	−0.576	−0.122	0.903
	North America	1.513	0.308	0.759
	Oceania	−1.327	−0.175	0.862
	South America	2.451	0.406	0.686

## Discussion

To the best of our knowledge, the present study is the first meta-analysis to compare male and female IGT performance within the healthy population. The pooled analysis revealed a sex-related difference in IGT performance across the 110 included studies, with males obtaining higher total net scores than females (UMD = 3.381; *p* < 0.001). Furthermore, the data resulted as significantly heterogenous; heterogeneity is a measure of inter-study variability in terms of reported outcomes. The levels of heterogeneity observed suggest that there is significant variability between the results obtained by individual studies that have compared male and female performance on the IGT. Interestingly, the results were not moderated by either mean sample age, publication year, sample size, study quality, type of monetary reward (i.e., real or not real), task version (i.e., computerized or manual), or region in which the study was conducted (in terms of geographical continent).

Individual studies had previously suggested that there may be a sex-related difference in decision-making, as measured by the IGT (for a review, see van den Bos et al., 2013). By pooling all existing literature from the past 20 years, this study was able to systematically assess any potential differences between men and women at a greater scale in order to draw more robust conclusions and quantify the size of this presumed difference. The meta-analysis revealed that males performed significantly better than females in terms of the total net score, choosing advantageous decks more often than women throughout the 100 trials. In practical terms, IGT performance may be considered an indication of the ability to weigh the odds that one is faced with and delay short-term gratification in order to achieve long-term rewards; this ability may have complex ramifications in day-to-day situations where an individual is asked to make a decision to optimize long-term prospects regardless of potential short-term negative consequences, such as in a work setting.

Several mechanisms may underlie a difference in decision-making between men and women. Firstly, the current literature seems to suggest that there may be a neural basis for predicting differences in decision-making between men and women, and that sex-related functional asymmetries in the VMPFC and amygdala may be at the forefront of the observed differences (Weller & Colleagues, 2010). The IGT seems to be a task predominantly associated with the right hemisphere (Christman et al., 2007; Naccache et al., 2005), and interestingly, the right hemisphere seems to be more involved in the decision-making process in men, whereas the opposite seems to be true for women. For example, emotional arousal has been found to enhance memory through the activation of the right amygdala in men, and through the left amygdala in women (Cahill et al., 2004). Additionally, lesion studies have found that men with VMPFC or right amygdala lesions and women with VMPFC or left amygdala lesions manifested social functioning and decision-making deficits; in contrast, the same deficits were not observed when the opposite hemispheres of the same areas were lesioned (i.e., left in men and right in women) (Tranel et al., 2005). Similar results were also observed in functional magnetic resonance imaging (fMRI) studies, in which more lateralized activations in the right hemisphere were observed in men during the IGT compared to women (Bolla et al., 2004). Taken together, these results seem to suggest that this proposed functional asymmetry may be partly responsible for the main finding of the present study.

Another hypothesis concerns a difference in sensitivity to wins and losses. Within the healthy population, the disadvantageous deck B is chosen almost as often as the advantageous deck D, suggesting that frequency of wins/losses may be more important than the actual size of the win/loss when performing the task (Lin et al., 2012). This phenomenon is known as the prominent deck B phenomenon and seems to be more pronounced in women, suggesting that they have a greater sensitivity to frequency of wins/losses than men (van den Bos et al., 2013). Furthermore, Garrido-Chaves and colleagues (2020) observed a greater amplitude of the Feedback-Related Negativity (FRN) component in women participating in the IGT. The FRN is a brain signal that usually peaks 260 ms after receiving unfavorable feedback and is particularly sensitive to losses following the presentation of an economic feedback. The authors therefore suggest that women may show greater sensitivity to perceived financial losses in the IGT (Garrido-Chaves et al., 2020).

Finally, Villanueva-Moya and Expósito (2021) highlight the relevance of sociocultural factors in women’s decision-making process. Their study revealed that when placed in a stereotype-threat condition, women make riskier and more disadvantageous decisions than men under the same conditions or women in non-stereotyped threat conditions; the same result is observed when in fear of a negative evaluation. The authors therefore emphasize the relevance of psychosocial variables that legitimize gender inequality in women’s decision-making process (Villanueva-Moya & Expósito, 2021).

Although all of these aforementioned mechanisms may be partly responsible in determining the observed result of a better male performance in the IGT, it is also important to note that, to the best of our knowledge, measurement invariance across sexes has not yet been assessed. It may be possible that men and women do not interpret the task in the same way, and thus must be considered one of the variables potentially contributing to this result.

The heterogeneity observed in the outcomes of the studies may be due to several variables that have been found to differentially impact the male and female decision-making process, such as stress, testosterone levels, trait anxiety, and the menstrual cycle. Specifically, higher cortisol levels in men are associated with a poorer performance, whereas an inverse relationship is observed in women, suggesting that women may make better long-term decisions than men under stress (Van den Bos et al., 2009). Furthermore, higher testosterone levels have been linked to riskier choices in both men and women; interestingly, this effect seems to be more pronounced in women (Stanton et al., 2011). Regarding trait anxiety, low and high levels of trait anxiety in men have both been associated with an impaired IGT performance; on the contrary, IGT performance in females seems to only be hindered by high levels of trait anxiety (de Visser et al., 2010). Finally, the phase of the menstrual cycle may affect women’s sensitivity to rewards, potentially influencing their performance on this task; for example, in the follicular phase, women seem to be more sensitive to the rewarding aspects of d-amphetamine than in the luteal phase (Justice & de Wit, 1999). All of these factors may have played a role in determining the high variability in terms of the outcomes reported by the individual studies.

Although many factors may play a role in determining sex-related differences in decision-making, moderator analysis showed that the results of the meta-analysis did not differ based on either mean sample age, publication year, sample size, study quality, type of monetary reward, task version, or region in which the study was conducted. A moderator is any variable that conditions the relationship between the two main variables (i.e., IGT performance and sex). In this particular case, none of the aforementioned variables moderated the main finding of a better male IGT performance. Regarding the variable mean age, the current meta-analysis may not be representative of all age ranges due to the exclusion of children and to the possible over-representation of studies conducted on university students. Moreover, although 20 years of data were included, the results did not differ based on the year of publication. The results obtained regarding the moderators “type of monetary reward” (i.e., real or fake money) and “task version” (i.e., computerized or manual) confirm and extend previous findings which suggest that differences in task versions and type of reward received do not significantly impact performance (Bowman & Turnbull, 2003; Bowman et al., 2005; Fernie & Tunney, 2006).

Finally, the analyses conducted to assess and adjust for any potential publication bias found no evidence of small-study effects, for which publication bias would have been considered one of the possible causes; small-study effects occur when the effects observed in smaller studies are different than those obtained in larger studies (Egger et al., 1997). Furthermore, when using a selection model approach to adjust the estimated effect for publication bias, the adjusted effect remained significant, albeit decreasing in size (adjusted UMD = 2.747; *p* = 0.010).

## Limitations

The present study has several limitations. Firstly, the selection process was limited to the past 20 years; thus, any study conducted prior to this time frame was excluded from the database search. Furthermore, despite identifying 11 grey literature studies that complied with the inclusion criteria, the necessary data was not available within the dissertations/articles themselves and the authors failed to respond to the data request made via e-mail; the present study therefore does not include data deriving from grey literature. Secondly, the study did not assess measurement invariance; thus, it may be possible that men and women simply do not interpret the task in the same way. Moreover, the inclusion criteria were restricted to articles that used the classic 100-trial IGT. Past research has also suggested that any sex-related difference in performance may only emerge as a difference in the learning curve, in that women tend to learn which decks to avoid later than men (van den Bos et al., 2007). It would therefore be interesting to explore whether the observed difference between males and females persists in longer versions of the task (e.g., 120-trial task). Furthermore, by only taking the net score into consideration, specific differences in terms of deck preferences, strategies, or phases of the task were not explored. For example, the IGT may be subdivided into an initial learning phase (i.e., the first 40 trials) and a second risk phase (i.e., the final 60 trials) to explore strategy development throughout the progression of the task. Previous studies have suggested that a sex-related difference may only emerge around the 60th trial of the task, implying that although both sexes initially consider all four decks equally, throughout the progression of the task men tend to learn to exclude the disadvantageous choices earlier than women (van den Bos et al., 2007). These results suggest that women tend to persist in the initial erroneous strategy of considering all four decks equally, rather than identifying and excluding the disadvantageous ones.

It is also important to highlight that the meta-analysis revealed significant heterogeneity, suggesting high variability between the results obtained by individual studies that have compared male and female performance on the IGT. In fact, despite of the significant result obtained, articles included in the present study often reported mixed results, suggesting that there are considerable discrepancies across studies’ outcomes. As displayed by the forest plot in Fig. 2, numerous studies failed to observe any sex difference in IGT performance, and, in several cases, even reported higher scores obtained by women.

## Conclusions

Limitations notwithstanding, this pooled analysis of the last 20 years of data concerning IGT performance in males and females highlighted a sex-related difference in IGT performance, with males obtaining higher scores than females. However, it also revealed significant variability and inconsistencies in the results of studies that measured IGT performance within the normal population.

These findings may have methodological and clinical implications. From a methodological point of view, a potential sex-related difference must be taken into account during the sampling process of any study which aims to use the IGT within its research, with particular emphasis on the various factors which may bring about individual differences in performance, as emphasized by the significant heterogeneity observed.

From a clinical perspective, it is particularly curious to note that sex differences have not been identified in other decision-making tasks, such as the Game of Dice task and the Balloon Analogue Risk task (van den Bos et al., 2013). It is reasonable to assume that the result that emerged from the present study may be due to the specific characteristics of this particular task, rather than decision-making in general. The IGT has been specifically built to mimic the experience of gambling and to simulate the perceived prospect of a financial gain/loss. Interestingly, males are also typically more likely than females to be either at-risk or problem gamblers (Merkouris et al., 2016). In fact, a recent study by Carneiro and colleagues (2020) found that males were 2.3 times more at risk of gambling exposure and 3.6 times more likely to experience gambling-related problems. Future research may want to explore whether there is a link between men performing better than women on the IGT and their known vulnerability to pathological gambling.

## Supplementary Information

Below is the link to the electronic supplementary material.

Supplementary file1 (PDF 85 KB)

Supplementary file2 (PDF 13 KB)

Supplementary file3 (PDF 205 KB)

## Data Availability

The data that supports the findings of this study is available at https://figshare.com/s/9e469dcd851937c51ce1.
