# Supplementary material for: The Iowa Gambling Task: Men and Women Perform Differently. A Meta-analysis
Source: Neuropsychol Rev. 2024 Mar 11;35(1):211–31. doi: 10.1007/s11065-024-09637-3 (PMC11965174; doi:10.1007/s11065-024-09637-3)
Supplement: Supplementary file 2 — Supplementary file2 (PDF 13 KB) [file 11065_2024_9637_MOESM2_ESM.pdf]

The Iowa Gambling Task: men and women perform differently. A meta-analysis
Neuropsychology Review

Ludovica Zanini*, Chiara Picano, Grazia Fernanda Spitoni
*Sapienza University of Rome (Department of Dynamic and Clinical Psychology, and Health Studies), Rome, Italy; ludovica.zanini@uniroma1.it

Online Resource 2: The Newcastle-Ottawa Scale

The Newcastle-Ottawa Scale used in the present study for quality assessment (maximum 7 stars).

**Selection (maximum 2 stars):**
Representativeness of the general population sample:<br>1) Truly representative of the average in the target population (all subject or random sampling). *<br>2) Somewhat representative of the average in the target population (non-random sampling). *<br>3) Selected group of users.<br>4) No description of the sampling strategy<br>***Sample size:***<br>1) Satisfactory (minimum 30).*<br>2) Not satisfatory.
**Measurement of the variables of interest (maximum 2 stars):**
1) The procedure is available or described. **<br>2) No description of the measurement procedure.
**Outcome (maximum 3 stars):**
Assessment of the outcome:<br>1) Detailed description. **<br>2) No description.<br>***Statistical test:***<br>1) The statistical test used to analyze the data is clearly described and appropriate, and the measurement of the association is presented, including confidence intervals and the probability level (p value). *<br>2) The statistical test is not appropriate, not described or incomplete.
