# Supplementary material for: The Iowa Gambling Task: Men and Women Perform Differently. A Meta-analysis
Source: Neuropsychol Rev. 2024 Mar 11;35(1):211–31. doi: 10.1007/s11065-024-09637-3 (PMC11965174; doi:10.1007/s11065-024-09637-3)
Supplement: Supplementary file 3 — Supplementary file3 (PDF 205 KB) [file 11065_2024_9637_MOESM3_ESM.pdf]

The Iowa Gambling Task: men and women perform differently. A meta-analysis
Neuropsychology Review

Ludovica Zanini*, Chiara Picano, Grazia Fernanda Spitoni
*Sapienza University of Rome (Department of Dynamic and Clinical Psychology, and Health Studies), Rome, Italy; ludovica.zanini@uniroma1.it

Online Resource 3: Quality Assessment

Details on the quality assessment indices for the retrieved studies.

Study	Selection		Measurement of variables of interest	Outcome		Total
	Truly/somewhat representative of population (*)	Sample size ≥30 (*)	Procedure is available/described (**)	Detailed description (**)	Statistical testing is appropriate and described (*)	
Alameda-Bailén et al., 2018	NO	YES	YES	YES	YES	6
Aloi et al., 2020	NO	NO	YES	YES	YES	5
Bangma et al., 2019	YES	YES	YES	YES	YES	7
Barnhart et al., 2019	NO	YES	YES	YES	YES	6
Birkàs et al., 2015	NO	YES	YES	YES	YES	6
Bolla et al., 2004	NO	NO	YES	YES	YES	5
Bonnaire et al., 2022	YES	YES	YES	YES	YES	7
Bouchard et al., 2012	NO	NO	YES	YES	YES	5
Brunell & Buelow, 2017 (Study 1)	NO	YES	YES	YES	YES	6
Brunell & Buelow, 2017 (Study 2)	NO	YES	YES	YES	YES	6
Brunell & Buelow, 2017 (Study 3)	NO	YES	YES	YES	YES	6
Buelow & Barnhart, 2017	NO	YES	YES	YES	YES	6
Buelow & Barnhart, 2018	NO	YES	YES	YES	YES	6
Buelow & Blaine, 2015	NO	YES	YES	YES	YES	6

Buelow & Brunell, 2020	NO	YES	YES	YES	YES	6
Buelow & Suhr, 2013	NO	YES	YES	YES	YES	6
Buelow & Suhr, 2014	NO	YES	YES	YES	YES	6
Buelow & Wirth, 2017 (Study 1)	NO	YES	YES	YES	YES	6
Buelow & Wirth, 2017 (Study 2)	NO	YES	YES	YES	YES	6
Buelow et al., 2013 (Study 1)	NO	YES	YES	YES	YES	6
Buelow et al., 2013 (Study 2)	NO	YES	YES	YES	YES	6
Buelow et al., 2015a	NO	YES	YES	YES	YES	6
Buelow et al., 2015b	NO	YES	NO	YES	YES	4
Burke et al., 2011	NO	YES	NO	YES	YES	4
Bø et al., 2016	YES	YES	YES	YES	YES	7
Casey & Cservenka, 2020	NO	YES	YES	YES	YES	6
Clay & Parker, 2018	NO	NO	YES	YES	YES	5
Crane et al., 2013	YES	YES	YES	YES	YES	7
Daurat et al., 2013	NO	NO	YES	YES	YES	5
Delazer et al., 2016	YES	NO	NO	YES	YES	4
Demaree et al., 2010	NO	YES	YES	YES	YES	6
Dingemans et al., 2019	YES	YES	YES	YES	YES	7
Dreves et al., 2020	NO	YES	YES	YES	YES	6
Dreyer et al., 2022	NO	YES	YES	YES	YES	6
Emery et al., 2020	YES	YES	YES	YES	YES	7
Farrell & Walker, 2019	YES	YES	YES	YES	YES	7
Favieri et al., 2022	NO	YES	YES	YES	YES	6
Fernandez et al., 2022	NO	NO	YES	YES	YES	5

Gescheidt et al., 2013	NO	NO	YES	YES	YES	5
Ghosh et al., 2021	NO	NO	YES	YES	YES	5
Giustiniani et al., 2019	NO	NO	YES	YES	YES	5
Gkintoni et al., 2017	NO	YES	YES	YES	YES	6
Gullo & Stieger, 2011	NO	YES	YES	YES	YES	6
Hart et al., 2010	NO	YES	YES	YES	YES	6
Hayes & Wedell, 2020a	NO	YES	YES	YES	YES	6
Hayes & Wedell, 2020b	NO	YES	YES	YES	YES	6
Heilman & Miclea, 2015	NO	YES	YES	YES	YES	6
Hulka et al., 2014	YES	YES	YES	YES	YES	7
Icellioglu, 2015	NO	YES	YES	YES	YES	6
Kashyap et al., 2013	NO	YES	NO	YES	YES	4
Kim et al., 2009	NO	YES	YES	YES	YES	6
Kobayakawa et al., 2008	NO	NO	YES	YES	YES	5
Kräplin et al., 2014	NO	YES	YES	YES	YES	6
Lage et al., 2013	NO	YES	YES	YES	YES	6
Lai et al., 2023	YES	YES	YES	YES	YES	7
Lake et al., 2020	YES	NO	YES	YES	YES	6
Lee et al., 2009	NO	YES	YES	YES	YES	6
Leonello & Jones, 2016	NO	YES	YES	YES	YES	6
León et al., 2020	NO	YES	YES	YES	YES	6
Linhartová et al., 2020	YES	YES	YES	YES	YES	7
Lovallo et al., 2014	YES	YES	YES	YES	YES	7
Lucas et al., 2021	NO	YES	YES	YES	YES	6
MacLaren et al., 2022	NO	YES	YES	YES	YES	6
Maddaluno et al., 2022	YES	YES	YES	YES	YES	7

Martín-Ríos et al., 2022	NO	YES	YES	YES	YES	6
Massar et al., 2014	NO	YES	YES	YES	YES	6
Maurage et al., 2018	NO	YES	YES	YES	YES	6
Merchán-Clavellino et al., 2019	NO	NO	YES	YES	YES	5
Meshi et al., 2019	YES	YES	YES	YES	YES	7
Miu et al., 2012	NO	YES	YES	YES	YES	6
Molins et al., 2021	NO	YES	YES	YES	YES	6
Moniz et al., 2016	NO	YES	YES	YES	YES	6
Müller et al., 2021	NO	YES	YES	YES	YES	6
Namba, 2021	NO	YES	YES	YES	YES	6
Nicholson et al., 2021 (Study 1)	NO	YES	YES	YES	YES	6
Nicholson et al., 2021 (Study 2)	NO	YES	YES	YES	YES	6
Obeso et al., 2021	YES	NO	YES	YES	YES	6
Olkoniemi et al., 2016	NO	YES	YES	YES	YES	6
Olson et al., 2016	YES	YES	YES	YES	YES	7
Oswald et al., 2015	YES	YES	YES	YES	YES	7
Ouerchefani et al., 2017	NO	YES	YES	YES	YES	6
Paz-Alonso et al., 2020	NO	NO	YES	YES	YES	5
Penolazzi et al., 2013	NO	YES	YES	YES	YES	6
Premkumar et al., 2010	YES	NO	NO	YES	YES	4
Psederska et al., 2021	YES	YES	YES	YES	YES	7
Runyon & Buelow, 2019	NO	YES	YES	YES	YES	6
Sánchez-Torres et al., 2013	YES	YES	NO	YES	YES	5
Sebri et al., 2021	YES	YES	YES	YES	YES	7

Seubert-Ravelo et al., 2021	NO	NO	YES	YES	YES	5
Shukla et al., 2019	NO	NO	YES	YES	YES	5
Simonovic et al., 2017	NO	NO	YES	YES	YES	5
Singh, 2016	NO	YES	YES	YES	YES	6
Siqueira et al., 2022	NO	YES	YES	YES	YES	6
Stinson et al., 2018	YES	YES	YES	YES	YES	7
Stoltenberg & Vandever, 2010	NO	YES	YES	YES	YES	6
Tarantino et al., 2021	NO	YES	YES	YES	YES	6
Tchanturia et al., 2012	NO	YES	YES	YES	YES	6
Valentini et al., 2017	NO	NO	NO	YES	YES	3
Vila-Rodriguez et al., 2013	NO	YES	YES	YES	YES	6
Villanueva-Moya & Expósito, 2021	NO	YES	YES	YES	YES	6
Vrshek-Schallhorn et al., 2013	NO	NO	YES	YES	YES	5
Webb et al., 2014	NO	YES	YES	YES	YES	6
Werner et al., 2013	NO	NO	YES	YES	YES	5
Yechiam & Telpaz, 2013 (Study 2)	NO	YES	NO	YES	YES	4
Yechiam et al., 2008	YES	NO	YES	YES	YES	6
Yechiam et al., 2016 (Study 1)	NO	YES	YES	YES	YES	6
Zhang et al., 2017	NO	YES	YES	YES	YES	6
Zhang et al., 2022	YES	NO	YES	YES	YES	7
Zouraraki et al., 2019	NO	YES	YES	YES	YES	6
Zouraraki et al., 2020	NO	YES	YES	YES	YES	6
